# Investigating the complementary value of discrete choice experiments for the evaluation of barriers and facilitators in implementation research: a questionnaire survey

**DOI:** 10.1186/1748-5908-4-10

**Published:** 2009-03-01

**Authors:** Debby van Helvoort-Postulart, Trudy van der Weijden, Benedict GC Dellaert, Mascha de Kok, Maarten F von Meyenfeldt, Carmen D Dirksen

**Affiliations:** 1 Department of Clinical Epidemiology and Medical Technology Assessment, University Hospital Maastricht, PO Box 5800, 6202 AZ Maastricht, the Netherlands; 2 Department of General Practice, University of Maastricht, PO Box 616, 6200 MD Maastricht, the Netherlands; 3 School for Public Health and Primary Care (CAPHRI), University of Maastricht, PO Box 616, 6200 MD Maastricht, the Netherlands; 4 Department of Business Economics, section Marketing Erasmus University Rotterdam, PO Box 1738, 3000 DR Rotterdam, the Netherlands; 5 Department of General Surgery, University Hospital Maastricht, PO Box 5800, 6202 AZ Maastricht, the Netherlands

## Abstract

**Background:**

The potential barriers and facilitators to change should guide the choice of implementation strategy. Implementation researchers believe that existing methods for the evaluation of potential barriers and facilitators are not satisfactory. Discrete choice experiments (DCE) are relatively new in the health care sector to investigate preferences, and may be of value in the field of implementation research. The objective of our study was to investigate the complementary value of DCE for the evaluation of barriers and facilitators in implementation research.

**Methods:**

Clinical subject was the implementation of the guideline for breast cancer surgery in day care. We identified 17 potential barriers and facilitators to the implementation of this guideline. We used a traditional questionnaire that was made up of statements about the potential barriers and facilitators. Respondents answered 17 statements on a five-point scale ranging from one (fully disagree) to five (fully agree). The potential barriers and facilitators were included in the DCE as decision attributes. Data were gathered among anaesthesiologists, surgical oncologists, and breast care nurses by means of a paper-and-pencil questionnaire.

**Results:**

The overall response was 10%. The most striking finding was that the responses to the traditional questionnaire hardly differentiated between barriers. Forty-seven percent of the respondents thought that DCE is an inappropriate method. These respondents considered DCE too difficult and too time-consuming. Unlike the traditional questionnaire, the results of a DCE provide implementation researchers and clinicians with a relative attribute importance ranking that can be used to prioritize potential barriers and facilitators to change, and hence to better fine-tune the implementation strategies to the specific problems and challenges of a particular implementation process.

**Conclusion:**

The results of our DCE and traditional questionnaire would probably lead to different implementation strategies. Although there is no 'gold standard' for prioritising potential barriers and facilitators to the implementation of change, theoretically, DCE would be the method of choice. However, the feasibility of using DCE was less favourable. Further empirical applications should investigate whether DCE can really make a valuable contribution to the implementation science.

## Background

There are numerous implementation strategies available that have proven to be at least moderately effective in bringing about change [1-3]. Current insight in implementation research is that the choice of implementation strategy should be guided by a diagnostic analysis that starts with describing the gap between current care and optimal care [4]. An important part of the diagnostic analysis is the identification of barriers and facilitators to change. Until now, studies identifying barriers and facilitators to change have been carried out using a combination of qualitative and quantitative methods, such as case-specific questionnaires, semi-structured in-depth interviews, focus group interviews, and non-participating observation [5]. These methods have some limitations. First, they generally yield many barriers, but do not provide information with respect to the relative importance, or prioritizing, of the barriers. Second, existing instruments hardly differentiate between barriers, and consequently may overestimate the importance of less important barriers and underestimate the importance of more important barriers. Third, these traditional methods have a non-compensatory character, which may be problematic because in decision processes concerning the implementation of change it is often the case that facilitators can partly compensate for barriers. These limitations associated with the methods that are currently applied in implementation research have revealed the need for an alternative research methodology for the evaluation of barriers and facilitators. Discrete choice experiments (DCE) to investigate preferences are relatively new in the health care sector, and may be of value in the field of implementation research. DCE is a stated preference method that presents individuals with a number of choices. Each choice consists of two or more hypothetical profiles, and for each choice, people are asked which profile they would choose. Forcing people to make choices and trade-offs is a big advantage of DCE over the methods that are currently applied in implementation research. For us, this is a strong motivation to introduce DCE in implementation research. If DCE proves to have a complementary value for the evaluation of barriers and facilitators, the choice of implementation strategy will be based on factors that more accurately reflect individuals' preferences and trade-offs, and the strategies will be tailored to the preferences of those concerned with the actual implementation.

The objective of our study was to investigate the complementary value of DCE for the evaluation of barriers and facilitators in implementation research. To meet this objective, we compared the results of a traditional questionnaire with the results of a DCE. Note that we use the term 'traditional' to refer to common, well-known research methodologies that are usually used in implementation research, in contrast to the DCE method that is new in the field of implementation research. We would expect *a priori *that DCE would overcome the previously mentioned limitations associated with using a traditional questionnaire. Especially, DCE makes it possible to gain insight into the relative importance of barriers to the implementation of change, and the trade-offs people make between barriers on the one hand and facilitators on the other hand. DCE therefore more closely reflects actual implementation decisions.

Respondents were expected to be unfamiliar with DCE, but also are actually involved in implementation processes and hence users of the results and responsible for getting the research findings into practice. Therefore, we asked respondents for their opinions about the feasibility of DCE. In particular, we asked for the completion time, the difficulty of the questions, and the appropriateness of DCE. The traditional questions are well-known, easy to administer and take little time to complete. Furthermore, the traditional method is often applied in implementation research, which is a strong indicator of its feasibility. DCE, on the other hand is new in implementation research, so we decided to evaluate only the feasibility and acceptance of this novel development.

## Methods

### Clinical subject

The clinical subject of this study was the implementation of the guideline for breast cancer surgery in day care. Breast cancer care causes a significant burden to the health care budget, which can mainly be attributed to surgical treatment [6], including hospitalization. In the University Hospital Maastricht, the Netherlands, an ambulatory surgery programme for breast cancer was developed and evaluated under the title of 'Breast pathology treated in day care: blessing or a curse?' [7]. Major objective of this programme was to reduce the length of hospital stay. The programme consists of a structured care system, including education and counselling, dedicated anaesthesia, active participation of the patient in her own treatment plan and in the decision to go home, and home nursing care. Traditionally, the surgical oncologist has been the specialist who analyses the type of breast pathology presented. Preventive procedures, early diagnosis of non-palpable lesions, breast conserving therapy, and lymph node sparing therapy have raised possibilities to reduce the burden of breast cancer surgery. The role of the breast care nurse was introduced to improve patient counseling. While the clinician's activities are limited to solving medical problems, the breast care nurse performs all coordinating tasks to create a programme that runs smoothly. Through these developments, diagnosis and treatment of breast pathology have gained a more multidisciplinary character. A decrease in the burden of surgery may limit the need for hospital-based supportive care. Similarly, this leads to a demand for strict coordination of the different steps and disciplines involved. Moreover, responsibilities for aspects of care need to be reallocated to other persons: from hospital-based supportive caregiver towards informal caregiver, from clinician to nurse specialist, from ward nursing staff to outpatient nursing staff, and from hospital-based nursing staff to home care nursing staff. Evaluation of the programme revealed that the breast cancer care programme was safe, reduced hospital stay, resulted in a high level of patient satisfaction, and resulted in lower cost of hospital stay. The guideline for breast cancer surgery in day care is currently being implemented in four other centres in the Netherlands as part of the implementation study entitled 'Introduction of a breast cancer care program in ultra short stay in four early adopter hospitals: implementation and evaluation'. Up till now, the Dutch Institute for Healthcare Improvement has not published the guideline for breast cancer surgery in day care officially.

### Discrete choice experiment

The DCE presented in this paper is described in detail elsewhere [8]. Therefore, we will now present the DCE method to the extent that the information is relevant for understanding of the present paper. For more theoretical background information about DCE, the interested reader is referred to Louviere *et al. *[9].

DCE is a stated preference method to measure preferences for products and services. It is based on the assumption that in general decisions are not based on a single criterion, but on several factors considered jointly [10]. In DCE jargon, these factors are called 'attributes'. A DCE consists of five stages: identifying the attributes of interest, assigning levels to the attributes, presenting profiles to individuals which involve different levels of the attributes, obtaining preferences for the profiles, and analyzing the responses.

A typical DCE starts with identifying the potential influential attributes. In our experiment, the attributes, being the potential barriers and facilitators to the implementation of the guideline for breast cancer surgery in day care, were selected on the basis of interviews with doctors and nurses who are all specialized in breast cancer surgery. In addition, we used the experience gained from the previously mentioned study 'Breast pathology treated in day care: blessing or a curse?' [7]. From the guideline for breast cancer surgery in day care that is composed of multiple recommendations, we selected the 12 key recommendations closely related to the surgical procedure (Appendix 1). Recommendations for the other parts of the programme were omitted. The 17 most frequently mentioned potential barriers and facilitators to the implementation of the 12 key recommendations were retained and included in the DCE as decision attributes.

Each of these attributes had two levels (Table 1). To handle these large numbers of potentially influential attributes, we used hierarchical information integration (HII). This is an alternative to standard discrete choice experiments when too many attributes are involved. HII relies on the assumption that, when confronted with complex decisions or evaluations involving numerous elements, people are able to divide a set of decision attributes that influence their choice behaviour into subsets that can be labelled in terms of high-order decision constructs, then evaluate each decision construct separately and aggregate their evaluations of each decision construct to choose between competing opportunities [11-13]. For details about our HII application, we refer to our Health Economics paper [8].

**Table 1:** Attributes and levels included in the discrete choice experiment

Attributes	Levels
1. day surgery unit	not available; available
2. breast care nursing staff	less than one full time equivalent; one full time equivalent or more
3. compensation	financial decline; no negative financial consequences
4. discharge criteria	not formulated; formulated
5. collaboration agreements with home care organizations	no; yes
6. patients/patient organizations	do not cooperate; cooperate
7. colleagues	do not cooperate; cooperate
8. management	do not cooperate; cooperate
9. ward nursing staff	do not cooperate; cooperate
10. expertise home care nurses	Insufficient; sufficient
11. written information after diagnosis	not available; available
12. preoperative counselling	not put in writing; put in writing
13. written information at discharge	not available; available
14. possibility to choose between day care and hospital admission	no; yes
15. patient satisfaction	remains the same; increases
16. status of the guideline	not published; published
17. time investment	more time-consuming; as much or less time

A full factorial design would require 512 profiles, too many for meaningful research. We therefore used fractional factorial designs based on an orthogonal main effects design. Profiles were paired into choice sets using a foldover design. This means that each of the profiles was combined with its 'foldover' profile. A foldover profile includes the exact opposite attribute levels of the original profile and, therefore, ensures a completely orthogonal design.

Next, respondents were presented with a series of pairwise profiles (choice sets) involving different levels of the attributes. Within each choice set, the profiles were labelled 'circumstances A' and 'circumstances B', and respondents were asked to choose between the two profiles for the implementation of breast cancer surgery in day care. Furthermore, respondents could select the 'neither' option if they did not find any of the two profiles acceptable for implementation. For an example of a discrete choice task see Additional file 1.

The choices were used to estimate the overall choice – or utility – model. Random parameters logit modelling was used to estimate this discrete choice model. We used the software package NLOGIT 3.0 (Econometric Software Inc.). The dependent variable was the choice alternative selected by the respondent. The independent variables were the 17 potential barriers and facilitators to the implementation of the guideline for breast cancer surgery in day care. We calculated the relative importance of the attributes as described in the literature [*e.g.*, [12]]. The 17 attributes were regarded as sources of respondents' utility, because the attributes are more or less important for successful implementation of this guideline. The overall utility may therefore be described as an evaluation of how attractive it is to implement the guideline for breast cancer surgery in day care, given the circumstances described by the attributes.

### Traditional questionnaire

To enable comparison of DCE with a traditional questionnaire, the same potential barriers and facilitators to the implementation of the guideline for breast cancer surgery in day care that were selected for the DCE were translated into statements (Appendix 2). Nine statements (1, 2, 4, 5, 11, 12, 13, 14, and 16) were phrased as preconditions that measure to what extent people believe that the specified requirements should be fulfilled to successfully implement change. The remaining eight statements (3, 6, 7, 8, 9, 10, 15, and 17) were phrased as expectations that measure to what extent respondents expect that the specified barriers will actually occur in their own hospitals. Respondents were asked to respond to the 17 statements on a five-point scale from one (fully disagree) to five (fully agree).

The responses to the traditional questionnaire were described in terms of means and medians. We also tested whether the responses were equally distributed across the response categories using Chi-Square tests. We used SPSS for Windows version 11.5 for the computations.

### Sample and data collection

Three groups of health care professionals were considered important for successful implementation of breast cancer surgery in day care, namely anaesthesiologists, surgical oncologists, and breast care nurses. The Dutch Society for Anaesthesiology and the Dutch Society for Surgical Oncology supplied the address files of their members. Questionnaires were sent by postal mail to anaesthesiologists and surgical oncologists, together with an informative letter to explain the background and aim of the study, and signed by the chair of the Dutch Society for Surgical Oncology. The distribution of questionnaires, including the informative letters, among breast care nurses took place through the chair of the special interest group Mammacare, which is part of the Society for Oncology Nurses. This group consists of nine members who represent the nine comprehensive cancer centre regions in the Netherlands. The questionnaires were distributed in the regions via these key contacts. Furthermore, the breast care nurses were encouraged to fill in the questionnaire by a message on the society's website.

A paper-and-pencil questionnaire was developed that consisted of five sections. Because the research presented in the present paper was part of a larger research project entitled 'Investigating the added value of conjoint analysis for the evaluation of barriers and facilitators in implementation studies: the case of breast cancer surgery in ultra-short stay', we will mention only those parts of the questionnaire that relate to the comparison between DCE and a traditional questionnaire. Respondents first completed some background questions, *i.e.*, age, sex, work experience, and mode of employment (salaried versus partnership). The next section included some questions about hospital characteristics. Then, respondents were asked to indicate their perceptions about the 17 potential barriers and facilitators in the 'traditional' way. In the final section, we first introduced the DCE thoroughly. We explained DCE by means of an example from everyday life. Then, respondents were asked to study the attributes and levels, and finally they completed a warm-up task with fill-in instructions. The attribute descriptions and levels, the warm-up task, and the fill-in instructions were presented on an insert. Respondents could take out this insert and re-read the information while evaluating the actual profiles. Following this introduction, respondents completed 14 actual discrete choice tasks. The section ended with three questions about the feasibility of DCE. First, respondents were asked how long it took to complete the discrete choice tasks. Secondly, they were asked to indicate on a scale from one (extremely difficult) to nine (extremely easy) the difficulty of the choice tasks. Finally, they were asked to briefly describe the appropriateness of DCE in implementation research. We calculated the mean number of minutes to complete the discrete choice tasks, and the mean score on the difficulty scale. The answers to the open question about the appropriateness of DCE were classified.

The questionnaire was pilot-tested beforehand. The aim of the pilot was to examine the respondents' understanding of the questionnaire. The pilot was designed as a think-aloud study; this involves respondents thinking aloud as they are completing the questionnaire. Eight respondents participated in the pilot test (three anaesthesiologists; two surgical oncologists; three breast care nurses). Based on the pilot test several changes were made to the layout of the questionnaire, and the wording of the instructions and questions.

## Results

### Response

We anticipated beforehand that health care professionals (especially physicians) would be difficult respondents to recruit for surveys. Therefore, we decided to approach all anaesthesiologists, all surgical oncologists and all breast care nurses. A total of 1,713 questionnaires were sent to 1,056 anaesthesiologists, 395 surgical oncologists, and 262 breast care nurses in five comprehensive cancer centre regions in the Netherlands. After six weeks, a reminder was sent. Data were collected between August 2006 and November 2006.

One hundred and seventy-four respondents returned the questionnaire (Table 2), resulting in an overall response of 10%. The response rates were 8%, 14%, and 13% for anaesthesiologists, surgical oncologists, and breast care nurses, respectively. The response rates differed statistically significant across these three professional disciplines (Chi-Square test; p < 0.001). We could not investigate whether selection bias has occurred because we lack information on the non-responders.

**Table 2:** Respondents' and hospital characteristics

Variable	N	Value
Professional discipline of respondents, %	174	
Anaesthesiologists	84	48
Surgical oncologists	56	32
Breast care nurses	34	20
Age, years	169	47 ± 8 (26–63)
Sex, % male	170	62
Work experience, years	157	13 ± 9 (0–32)
Employment, %	133*	
Salaried	57	43
Partnership	73	55
Both	3	2
Presence of an outpatient breast clinic, %	163	96
Availability of breast care nurses, fulltime equivalent	88	2.1 ± 1.4 (0–8)
Setting, %	96	
Ambulatory		25
24-hour stay		27
Admission (> 24 hours)		48
Presence of a day surgery unit, %	168	94

*Question not applicable to breast care nurses.

### Discrete choice experiment

Of 174 respondents who returned the questionnaire, 18 did not answer the discrete choice tasks. As a result, the responses of 156 respondents could be analyzed. Nearly all attributes were significant at the 1% level, implying that these attributes are relevant to the implementation of the guideline for breast cancer surgery in day care. Only the coefficient for time investment (p = 0.174) was not significant.

The relative importances of the attributes (Table 3) show that respondents' choices were influenced most strongly by the attribute 'cooperation of colleagues'. Therefore, whether or not colleagues would assist in implementing the guideline is the most important factor for health care professionals when they think of breast cancer surgery in day care. Also, the cooperation of the ward nursing staff and management was considered highly important. Cooperation of patients and patient organizations was considerably less important. Time investment, status of the guideline, and patient satisfaction were least influential.

**Table 3:** Relative attribute importance (DCE)

Attributes	RI
Cooperation colleagues	12. 8500 (1)
Cooperation ward nursing staff	10. 2877 (2)
Cooperation management	9. 5832 (3)
Compensation	8. 0656 (4)
Day Surgery Unit	7. 4579 (5)
Breast care nursing staff	5. 8002 (6)
Expertise home care nurses	5. 5743 (7)
Collaboration agreements with home care organizations	6. 1716 (8)
Cooperation patients/patient organizations	4. 8736 (9)
Preoperative counselling	5. 1813 (10)
Written information after diagnosis	5. 0234 (11)
Discharge criteria	3. 8478 (12)
Written information at discharge	5. 4884 (13)
Possibility to choose between day care and hospital admission	4. 3883 (14)
Patient satisfaction	4. 1348 (15)
Status of the guideline	1. 2989 (16)
Time investment*	0. 0271 (17)

DCE: discrete choice experiment.

RI: relative attribute importance; ranking is in parentheses.

*Attribute importance is only illustrative as the coefficient for time investment was not statistically significant.

### Traditional questionnaire

Table 4 presents the results of the traditional questionnaire. Statements related to preconditions and statements related to expectations are presented separately. The rankings of the responses are based on decreasing means. The most striking finding was that the responses to the statements hardly differentiated between barriers. This is particularly true when the median values are considered. Respondents considered the availability of patient information brochures after the diagnosis and at discharge, as well as clear-cut criteria for discharge as the most important requirements for successful implementation of the guideline for breast cancer surgery in day care. The presence of a Day Surgery Unit was considered the least important precondition.

**Table 4:** Health care professionals' perceptions about potential barriers and facilitators to the implementation of the guideline for breast cancer surgery in day care

Statement	Mean ± SD	Median (range)
Preconditions:		
Written information after diagnosis	4.30 ± 0.54	4; 2–5
Discharge criteria	4.18 ± 0.56	4; 2–5
Written information at discharge	4.08 ± 0.62	4; 2–5
Status of the guideline	4.05 ± 0.69	4; 2–5
Preoperative counselling	4.05 ± 0.69	4; 2–5
Breast care nursing staff	4.02 ± 0.73	4; 2–5
Collaboration agreements with home care organizations	3.93 ± 0.77	4; 1–5
Possibility to choose between day care and hospital admission	3.87 ± 0.86	4; 2–5
Day Surgery Unit	3.73 ± 0.94	4; 1–5

Expectations:		
Cooperation ward nursing staff	3.88 ± 0.52	4; 2–5
Cooperation patients/patient organizations	3.86 ± 0.48	4; 2–5
Cooperation colleagues	3.81 ± 0.53	4; 2–5
Cooperation management	3.81 ± 0.68	4; 1–5
Compensation	3.75 ± 0.86	4; 1–5
Expertise home care nurses	3.73 ± 0.83	4; 1–5
Patient satisfaction	3.52 ± 0.81	4; 1–5
Time investment	2.82 ± 0.90	3; 1–5

SD: standard deviation.

Respondents indicated on a 5-point scale from 1 (fully disagree) to 5 (fully agree) to what extent they agreed with the 17 statements.

The ranking of the expectations reflects to what extent each of the potential barriers to the implementation of the guideline for breast cancer surgery in day care is expected to actually become a barrier in respondents' own hospitals. Table 4 shows that respondents were, on average, most hopeful about the cooperation of those concerned with the implementation of the guideline, *i.e.*, the ward nursing staff, patients and patient organizations, colleagues, and the management. Working according to the guideline is not expected to be more time-consuming because respondents, on average, neither agreed nor disagreed with the concerning statement (Appendix 2, statement 17). This suggests that respondents do not perceive time as a major barrier to the implementation of the guideline. For all 17 statements, the responses were not equally distributed across the response categories (Chi-Square tests; p < 0.001).

### Feasibility of DCE

The number of minutes to complete the choice tasks was on average 25.5 ± 14.7 minutes. The completion time was 23.7 ± 15.4 minutes for anaesthesiologists, 24.9 ± 12.8 minutes for surgical oncologists, and 30.5 ± 14.9 minutes for breast care nurses. The three disciplines did not differ significantly from each other (ANOVA; p = 0.083).

The mean difficulty score was 4.8 ± 2.3 on a scale from one to nine. Mean score was 4.3 ± 2.2 for anaesthesiologists, 5.5 ± 2.2 for surgical oncologists, and 4.8 ± 2.3 for breast care nurses. The three disciplines differed significantly from each other (ANOVA; p = 0.023), *i.e.*, anaesthesiologists found the discrete choices significantly more difficult than the surgical oncologists (Bonferroni; p = 0.018).

We classified the answers to the open question about the appropriateness of DCE into three main categories. Twenty-six respondents found DCE an appropriate method, and 63 respondents thought that the method is inappropriate. Forty-five respondents did not explicitly state whether or not they think it is an appropriate method. These respondents did not respond to the methodology but instead they focussed on the clinical subject; other respondents answered: 'I do not know (or I doubt) whether this is an appropriate method' or 'I have no opinion'. Twenty-two respondents did not complete the question. The 63 respondents who judged DCE inappropriate gave in total 85 reasons for their opinion. We subdivided these reasons into seven subcategories. Table 5 shows that 'too difficult' was by far the most frequently mentioned reason (51%).

**Table 5:** Why respondents think DCE is an inappropriate method

Reason	Frequency
Too difficult	43

Too time-consuming	13

Boring/irritating/unpleasant	10

Unrealistic/illogical	7

Quality of results/data analysis too difficult	6

All attributes are important/circumstances should be optimal	4

Degree of abstraction	2

Total	85

Of 156 respondents, 63 thought that the DCE method is inappropriate. These 63 respondents gave in total 85 reasons.

## Discussion

To the best of our knowledge this is the first application of DCE in implementation research. We used DCE to identify barriers and facilitators to the implementation of the guideline for breast cancer surgery in day care. The objective of our study was to investigate the complementary value of DCE for the evaluation of barriers and facilitators in implementation research. To meet this objective we compared the results of a traditional questionnaire with the results of a DCE. In addition, we asked respondents for their opinions about the feasibility of DCE.

Neither DCE nor a traditional questionnaire was considered the 'gold standard' for identifying potential barriers and facilitators to the implementation of breast cancer surgery in day care. The reason why we conducted this study was that we would expect that DCE would provide implementers with more specific information to better fine-tune the implementation strategies. The results of a DCE provide implementation researchers and clinicians with a relative attribute importance ranking that can be used to prioritize potential barriers and facilitators to change. Prioritizing is useful to tailor the implementation strategies to the specific problems and challenges of a particular implementation process. Furthermore, the DCE method makes it possible to gain insight into the trade-offs people make between barriers to change on the one hand and facilitators on the other hand.

The traditional questionnaire was made up of statements that respondents answered on a five-point scale ranging from one (fully disagree) to five (fully agree). The questionnaire included both statements related to preconditions and statements related to expectations. Both kinds of statements cannot be compared to each other because the interpretation of the responses is considerably different. Statements phrased as preconditions measure whether the specified requirements should be fulfilled to successfully implement change. Statements phrased as expectations measure whether the specified barriers are expected to actually occur in respondents' own hospitals. Although the extent to which respondents agreed with the statements about the preconditions might be interpreted as a measure of the importance respondents attach to each of the requirements, it does not provide information about the relative importance of the barriers and facilitators and the trade-offs people make. Hence, a traditional questionnaire cannot easily be used to identify the most crucial barriers and facilitators to successful implementation and to leave aside the relatively unimportant ones. Furthermore, we must interpret the results of the traditional questionnaire with caution because the responses to the statements hardly differed from each other. One could argue that essentially respondents agreed with all statements to the same extent, which makes ranking less useful.

The results of our DCE and traditional questionnaire would possibly lead to different implementation strategies. This confronts those who are involved in implementation processes with serious difficulties, because we do not know which method is the best one. There are three major differences between DCE and the traditional questions that might challenge the comparability of the results. First, DCE is based on random utility theory that assumes that an individual acts rationally and always chooses the alternative with the highest level of utility. In our experiment, the 17 attributes (the independent variables in the utility function) are regarded as sources of respondents' utility because the attributes are more or less important for successful implementation of the guideline for breast cancer surgery in day care. The overall utility may therefore be described as an evaluation of how attractive it is to implement the guideline for breast cancer surgery in day care given the circumstances as described by the attributes. DCE is therefore a preference-based method in contrast with the traditional questionnaire that has no theoretical underpinnings. Second, the regular questions are more likely to measure the perceived barriers and facilitators that may not reflect the actual barriers and facilitators. The results of a DCE are likely to give a more objective view of the factors that are important for successful implementation. When responding to the regular questions, respondents are generally inclined to refer to the circumstances in their own hospitals ('which barriers would we encounter in our hospital?'). Whether respondents really answer the regular questions with their own hospitals' situations in mind depends to a great extent on the wording of the questions, which is not standardized. Discrete choice tasks present subjects with hypothetical scenarios; hence there is no link between the choices and subjects' current clinical practice. Third, in a DCE people are forced to make choices and trade off attributes, which is closer to the decision-making context in reality than the regular questions.

We encountered a low overall response rate of 10%. In accordance with Sitzia and Wood [14], sending reminders did little to increase the response rate. Response rates for mail surveys are approximately 62% [15]. Large variation is reported for response rates for DCE in the health care literature, ranging from 18% [16] to 98% [17]. We do not know if, to what extent, and how the low response has influenced the results. In the current study, there are four possible explanations for the low response. First, we investigated a complex multi-faceted health care decision – the implementation of the guideline for breast cancer surgery in day care – that moreover involves multiple professional disciplines. In implementation research, it is important to take into consideration this multidisciplinary nature of many health care decisions because different groups of health care professionals may express different opinions, interests, and preferences. Hence, the potential barriers and facilitators to the implementation of the guideline need not necessarily be the same for anaesthesiologists, surgical oncologists, and breast care nurses. To facilitate comparison between these groups, we developed an identical questionnaire for all three disciplines. Although the advantages of this approach are obvious, a disadvantage is that we could not completely fine-tune the questions to the specific circumstances of each professional group. Therefore, respondents may have considered the questions too broad and not fully applicable to their own situations. In particular, anaesthesiologists were expected to respond suboptimally because this group of health care professionals is less closely associated with the subject of the survey compared to surgical oncologists and breast care nurses. Respondents may thus have had less affinity with the questions. A second explanation for the low response may be the use of self-complete postal questionnaires. Other more personal data collection methods are available, and telephone or face-to-face interviews might well have increased the response rate, though at much higher costs. The phenomenal growth of internet technology in recent years has prompted many health care researchers to find ways of using this technology to communicate more effectively. Braithwaite *et al. *[18] examined whether internet-based surveys among health professionals can offer a valid alternative to traditional survey methods. They performed a systematic review of published internet-based surveys among health professionals and found response rates ranging from 9% to 94%. The authors also conducted an internet-based survey among general practitioners that explored attitudes about using an internet-based decision support system for the management of familial cancer. They achieved a response rate of 52.4% after five e-mail reminders. Brown and Kittleson [19] compared the response rates from an e-mail survey (43%) and a web-based survey (48%), and found no statistically significant differences between the two groups. An online survey among chiropractors yielded a response rate of 35.8% [20]. As online surveys become more feasible for more populations, it is worth considering the use of e-mail or the web as data collection tools in future research.

Third, the clinical subject may have been too complex to introduce DCE in implementation research. The implementation of the guideline for breast cancer surgery in day care is a complex process that involves changes on the organizational and management level, as well as the level of health care professionals and patients. The complexity of the clinical subject furthermore required that we had to include large numbers of potentially influential attributes. Therefore, we used HII, which is a more complex alternative to standard DCE.

Fourth, the questionnaire included both the traditional questions and the discrete choice tasks. All respondents first answered the traditional questions and then the discrete choice tasks. We do not know whether the response would have been better if respondents had been offered only the traditional questions or only the discrete choices. Several studies have shown that response rate is not correlated to questionnaire length [14,15,21]. Intuitively, we would nevertheless expect a higher response rate if the questionnaire included only the traditional questions for three reasons. First, our questionnaire was lengthy, and obviously completing only the traditional questions takes less time. Second, because respondents were likely to be unfamiliar with DCE, the discrete choice tasks were introduced thoroughly. Yet, this extensive introduction and warm-up task with fill-in instructions required a lot of reading, and thus time and motivation. Mean completion time of the discrete choice tasks was more than 25 minutes. Third, discrete choice tasks are without doubt more cognitively demanding than the traditional questions. The mean difficulty score was 4.8 on a scale from 1 to 9, which means 'somewhat difficult' to 'not difficult/not easy'. Eighteen respondents did not answer the discrete choice tasks. Although we did not systematically investigate why these respondents did not complete the choice tasks, we suppose that the two main reasons are the complexity of the DCE and the length of the questionnaire. We do not expect that these 18 respondents would have completed the DCE if the choices were presented before the traditional questions. The answers to the open question about the appropriateness of DCE are in support of this, and suggest doubt about the feasibility of DCE.

A considerable variety exists in the design and analysis of studies investigating barriers and facilitators to the implementation of change. In most cases, the analysis is constrained to descriptive statistics. Because of limitations associated with the methods typically applied in implementation research (see introduction), uncertainty exists with respect to the most useful research methodology. DCE should theoretically overcome the limitations of traditional methods, and provide implementers with more specific information, *i.e.*, insight into the trade-offs people make and relative attribute importance. Because DCE probably reflects actual implementations decisions more closely, it is expected that the implementation strategies will become more tailored to the actual preferences, needs, and wishes of those who are involved in the actual implementation process. It is furthermore expected that this will increase the cost-effectiveness of implementation strategies. So, on theoretical and conceptual grounds it can be suggested that DCE should be considered the reference standard in our study [8]. From a practical point of view, however, our study revealed that DCE could not entirely fulfil the role of 'gold standard'. In brief, respondents considered the method too difficult and too time-consuming, which may partly explain the low response. The feasibility of any method is – at least partly – dependent on the study context such as the target respondents, study design, clinical subject, and data collection method. Further empirical applications should investigate whether DCE can really make a valuable contribution to the implementation science.

## Conclusion

DCE was proposed as a tool to identify potential barriers and facilitators to the implementation of change. The results of a DCE and a traditional questionnaire would probably lead to different implementation strategies. Although there is no 'gold standard' for prioritising potential barriers and facilitators to the implementation of change, theoretically, DCE would be the method of choice. However, the feasibility of using DCE was less favourable; respondents considered the method too difficult and too time-consuming, which may – at least partly – explain the low response.

## Competing interests

The authors declare that they have no competing interests.

## Authors' contributions

DHP has made substantial contributions to the design of the study and planning of the work that led to the manuscript, the acquisition, analysis and interpretation of data, and has been involved in drafting and critically revising the manuscript for important intellectual content. TW has made substantial contributions to the conception and design of the study, the acquisition, analysis and interpretation of data, and has been involved in drafting and critically revising the manuscript for important intellectual content. BGCD has made substantial contributions to the conception and design of the study, the analysis and interpretation of data, and has been involved in drafting and critically revising the manuscript for important intellectual content. MK has made substantial contributions to the design of the study, and has been involved in critically revising the manuscript for important intellectual content. MFM has made substantial contributions to the conception and design of the study, and has been involved in critically revising the manuscript for important intellectual content. CDD has made substantial contributions to the conception and design of the study and planning of the work that led to the manuscript, the acquisition, and interpretation of data, and has been involved in drafting and critically revising the manuscript for important intellectual content. All authors have read and approved the final submitted version of the manuscript.

## Appendix 1: Key recommendations

1. In hospital at least one breast cancer nurse should be available at all times

2. The surgical oncologist and breast cancer nurse are both responsible for information about diagnosis and treatment options (preoperative counselling)

3. Oral counselling after the diagnosis is supported by written information

4. Patients should be visited by the surgeon preoperatively on the day of surgery

5. Patients should be visited by the breast cancer nurse preoperatively on the day of surgery

6. Patients should receive information from the surgeon before discharge

7. Patients should receive information from the anesthesiologist before discharge

8. Patients should be visited by the breast care nurse before discharge

9. Patients who are scheduled for day care surgery should be allowed to decide postoperatively in favour of an admission into hospital

10. Oral counselling at discharge is supported by written information

11. Patients are discharged based on clear-cut discharge criteria

12. Specialized home care is available for a period of time after surgery

## Appendix 2: Statements included in the traditional questionnaire*

1. Implementation of the guideline for breast cancer surgery in day care requires that a Day Surgery Unit is available

2. In my opinion working according the guideline requires the availability of a breast cancer nurse staff the size of at least one full time equivalent

3. I do not expect my income declines if the guideline for breast cancer surgery in day care are implemented

4. In my opinion working according the guideline requires that clear-cut discharge criteria are formulated

5. In my opinion working according the guideline requires that collaboration agreements are made with home care organizations

6. I expect that patients/patient organizations cooperate in applying the guideline

7. I expect that colleagues cooperate in applying the guideline

8. I expect that management cooperates in applying the guideline

9. I expect that ward nursing staff cooperates in applying the guideline

10. I expect home care nurses have enough expertise to provide postoperative care at home

11. In my opinion working according the guideline requires the availability of written information that supports the oral counselling after the diagnosis

12. In my opinion working according the guideline requires that the content of the preoperative counselling is put in writing

13. In my opinion working according the guideline requires the availability of written information that supports the oral counselling at discharge

14. In my opinion working according the guideline requires that patients who are scheduled for day care surgery are allowed to decide postoperatively in favour of an admission into hospital

15. I expect that patient satisfaction increases as a result of implementation of the guideline

16. I think it is important that the guideline is nationally supported, published and dispersed

17. I expect working according the guideline is more time-consuming for me as compared to the current situation

* Statements 1, 2, 4, 5, 11, 12, 13, 14, and 16 were phrased as preconditions; statements 3, 6, 7, 8, 9, 10, 15, and 17 were phrased as expectations.

## Supplementary Material

Additional file 1 **Example of a discrete choice task**. The table shows an example of a discrete choice task Click here for file
