# Supplementary material for: Investigating the complementary value of discrete choice experiments for the evaluation of barriers and facilitators in implementation research: a questionnaire survey
Source: Implement Sci. 2009 Mar 1;4:10. doi: 10.1186/1748-5908-4-10 (PMC2654421; doi:10.1186/1748-5908-4-10)
Supplement: Additional file 1 — Example of a discrete choice task. The table shows an example of a discrete choice task [file 1748-5908-4-10-S1.doc]

**Additional file 1: Example of a discrete choice task**

|  | Circumstances A | Circumstances B |
| --- | --- | --- |
| *Organization* |  |  |
| 1. Day surgery unit | Not available | Available |
| 1. Breast care nursing staff | Less than one full time equivalent | One full time equivalent or more |
| 1. Compensation | No negative financial consequences | Financial decline |
| 1. Discharge criteria | Formulated | Not formulated |
| 1. Collaboration agreements with home care organizations | No | Yes |
| Please give your opinion about *Organization* | ++++  +++  ++  +  0  -  - -  - - -  - - - - | ++++  +++  ++  +  0  -  - -  - - -  - - - - |
| *Cooperation partners* | - - | ++ |
| *Patient centeredness of care* | - - | ++ |
| *Status of the guideline* | Published | Not published |
| *Time investment* | As much or less | More time-consuming |
| Which circumstances would you choose? | Circumstances A | Circumstances B |
| Neither (no implementation of breast cancer surgery in day care) | | |
